# Recent Research Advances in the Application of Deep Eutectic Solvents for the Chemical Processes of the Nuclear Fuel Cycle

**DOI:** 10.3390/molecules31071107

**Published:** 2026-03-27

**Authors:** Zimo Wang, Liyang Zhu, Yan Zhang, Suliang Yang, Shengdong Zhang

**Affiliations:** Department of Radiochemistry, China Institute of Atomic Energy, Beijing 102413, China; wangzimowhu@outlook.com (Z.W.); zhangyan_fh@ciae.ac.cn (Y.Z.); ysl79@cnncmail.cn (S.Y.)

**Keywords:** deep eutectic solvents, actinides, radioactive nuclides, extraction, dissolution

## Abstract

As a new class of green functional liquids, deep eutectic solvents (DESs) have attracted increasing attention as alternatives to conventional solvents, such as mineral acids, organic solvents and ionic liquids (ILs), in nuclear chemistry. Owing to their low cost, easy preparation, structural tunability, and adjustable physicochemical properties, DESs provide unique solvation and coordination environments that enable various applications. This review summarizes recent research advances in the application of DESs for the chemical processes of the nuclear fuel cycle. Particular emphasis is focused on dissolution, extraction and separation, electrochemical deposition and redox processes, radionuclide capture, decontamination and detection. This review highlights the fundamental advantages and current limitations of DES-based systems and outlines future trends.

## 1. Introduction

The chemical processes of the nuclear fuel cycle are pertinent to fuel preparation and processing, spent fuel reprocessing, radioactive waste treatment, and the recovery of strategic nuclear materials. Conventional technologies for the dissolution, separation, and purification of relevant elements frequently use concentrated mineral acids and volatile organic solvents. Although these approaches have been successfully implemented at the industrial scale, they suffer from intrinsic drawbacks, including high corrosivity, solvent volatility, high waste generation, potential secondary radioactive waste generation, low efficiency and stringent safety requirements. With the continuous expansion of nuclear energy and increasing emphasis on sustainable development, there is a growing demand for greener, safer, and more adaptable solvent systems capable of addressing these long-standing challenges [1,2]. In this context, deep eutectic solvents (DESs) have emerged as a promising class of alternative solvents owing to their low cost, simple preparation, tunable physicochemical properties, high efficiency and improved environmental compatibility [3,4,5,6]. DESs are usually formed by hydrogen bonding interactions between hydrogen bond donors (HBDs) and hydrogen bond acceptors (HBAs), and this hydrogen bonding effect can produce a significant eutectic effect, reducing the melting point of the mixture, thereby forming a stable liquid phase [7]. Unlike ionic liquids (ILs), which often require complex synthesis and purification processes, DESs are typically prepared by simply mixing commercially available, low-cost raw materials under mild conditions without the need for tedious purification steps. This inherent simplicity of preparation, coupled with their low cost, make DESs highly attractive for large-scale industrial applications. The physicochemical properties of DESs are highly tunable, a key advantage that sets them apart from many conventional solvents. By adjusting the type of HBD and HBA components, their molar ratio, and even the addition of small amounts of water, properties such as the viscosity and acidity can be tailored to meet the specific requirements of different radiochemical processes [8,9,10]. For instance, hydrophilic DESs can be designed for solid–liquid extraction and dissolution processes, while hydrophobic DESs (HDESs), characterized by low water solubility and the formation of a distinct organic phase in aqueous systems, are suitable for liquid–liquid extraction or the selective dissolution of non-polar species [11]. Additionally, most DES systems exhibit low toxicity, low volatility, high efficiency and biodegradability, which significantly reduce environmental pollution and the generation of waste compared to traditional organic solvents and concentrated mineral acids.

Furthermore, the unique microsolvation effect of DESs is defined as a local microscopic structure formed by HBDs and HBAs through intermolecular hydrogen bonding, which regulates the solvation state, coordination configuration, and interaction energy of metal ions. This effect can significantly affect the speciation of metal ions, the kinetics of oxidation–reduction reactions, and the coordination behavior of ligands. This property makes DESs suitable for the dissolution of oxides, the control of valence states of actinides, the separation of actinides/lanthanides, and the electrochemical deposition of nuclear materials [12,13]. This feature provides DESs with broad application prospects across various stages of the nuclear fuel cycle.

Beyond nuclear-related processes, DESs have also demonstrated significant potential in metal recovery from lithium-ion batteries (LIBs) and the recycling of strategic metals such as rare earth elements, a rapidly growing area of resource recycling that aligns with global sustainability goals [14,15]. Despite rapid progress, the application of DESs in nuclear chemistry remains at an early stage, and existing studies are often fragmented, focusing on individual elements or isolated processes. Key issues such as the relationship between structure and performance, limitations arising from high viscosity, radiation stability under realistic conditions, and techno-economic feasibility have yet to be systematically addressed. An assessment of recent advances is necessary to clarify the current state of the field and to guide future research toward practical implementation. In this review, recent research advances in the application of DESs for the chemical processes of the nuclear fuel cycle are systematically summarized. The emphasis is placed on the DES-assisted dissolution of actinides, extraction and separation processes relevant to the nuclear fuel cycle, as well as electrochemical processing [3,16] and emerging applications in radioactive waste management [17,18,19,20] and radionuclide detection. By correlating DES composition with metal speciation and process performance, this review aims to highlight both the advantages and limitations of DESs and to provide insights into their future role in developing greener and more sustainable nuclear fuel cycle technologies.

## 2. Properties and Classification of DESs

Based on the nature of their components, DESs are commonly classified into five types (Type I-V) [11]. They can be hydrophobic [21,22,23], amphiphilic [24] or hydrophilic depending on the type of components. This classification scheme reflects the effects of different compositions on the properties of DESs.

Early DES systems (Type I and Type II) are primarily based on quaternary ammonium salts combined with metal halides or hydrated metal salts and are often sensitive to moisture [8,12,13,25]. Type III DESs, composed of quaternary ammonium salts and organic HBDs, represent a widely investigated category due to their structural diversity, low toxicity, and excellent tunability. The first example of DESs, classified as Type III DESs, was reported by Abbott et al. [26], using choline chloride (ChCl) and urea in a 1:2 molar ratio. In contrast, Type IV DESs, in which metal salts directly participate as eutectic components, exhibit distinct coordination environments and redox properties, making them particularly attractive for electrochemical processes and metal dissolution. Type IV DESs, composed of hydrated salts (lithium nitrate, cerium nitrate, gadolinium nitrate) and urea, have extremely high polarity [27]. Type V DESs consist exclusively of nonionic compounds and are typically hydrophobic, enabling efficient extraction [3,5,28] of non-polar species and biphasic separations. The classification presented in Table 1 provides a structural framework for understanding the relationship between DES composition and functionality.

The hydrophilic nature and structural versatility of Type III DESs make them ideal candidates for dissolution and solid–liquid extraction processes. In contrast, the hydrophobic character of Type V DESs is particularly suitable for liquid–liquid extraction. Meanwhile, Type IV DESs, with metal ions integral to their structure, offer unique coordination environments advantageous for electrochemical applications. Building upon this foundation, the following sections will explore the specific applications of DESs in detail.

## 3. Dissolution

The dissolution of actinide oxides (e.g., UO_2_, PuO_2_) is a critical first step in spent nuclear fuel reprocessing, traditionally requiring aggressive hot nitric acid. DESs offer a milder, alternative dissolution pathway, often without generating volatile or hazardous by-products.

### 3.1. Dissolution of Uranium Oxides

The dissolution of uranium oxides (UO_x_) is the first step in spent nuclear fuel reprocessing, traditionally accomplished by using hot, concentrated nitric acid. This method, while effective, raises significant environmental, safety, and corrosion concerns due to the generation of nitrogen oxide gases and large volumes of acidic waste. Although ILs offer excellent performance as alternative solvents, their high cost hinders large-scale industrial application. In contrast, DESs are inherently cost-effective and readily accessible. They also exhibit comparable or superior performance in uranium oxide dissolution without requiring additional mineral acids or releasing harmful volatiles. Therefore, DESs represent a more practical green solvent alternative for this critical process.

DESs composed of p-toluenesulfonic acid monohydrate (PTSA) and ChCl, as well as DESs composed of ChCl and malonic acid (MA), can effectively dissolve uranium oxides (UO_3_, UO_2_ and U_3_O_8_) [29]. Among them, PTSA:ChCl (1:2) exhibits the highest dissolution efficiency. A 100 mg sample of UO_2_ was completely dissolved in 2 mL of DES within 4 h, corresponding to a solubility of about 84 mg/mL. UV-Vis analysis indicated oxidation of UO_2_ to U(VI) during dissolution. Notably, luminescence spectroscopy revealed that the DES seemed to stabilize uranium-oxygen single bonds instead of multiple bonds so that U(VI) can exist either as UO_6_^6−^ or UO_4_^2−^ rather than as the conventional uranyl ion (UO_2_^2+^), highlighting the unique speciation control offered by the DES environment. Similarly, another DES (ChCl:MA = 1:1) based on ChCl can also directly dissolve various uranium matrices (UO_3_, UO_2_, U_3_O_8_, uranium metal, etc.) in a single step under mild heating, without added acids or harmful gas release. Uranium forms a [UO_2_(MA)_2_(H_2_O)_2_]^2+^ complex with equatorial coordination of MA and H_2_O, exhibiting a binding constant (log K = 1.20) higher than that of common weakly coordinating inorganic anions (Cl^−^, NO_3_^−^, and ClO_4_^−^), which facilitates dissolution and retention [30].

Imidazolium-based DESs have also been explored. Srivastava et al. [31] investigated the dissolution of UO_2_ and UO_3_. The solubility of UO_3_ and UO_2_ in the DES (1-decyl-3-methyl imidazolium bromide (C_10_minBr):MA) and the DES (C_10_minBr:diglycolic acid) is approximately 4.5 to 5 mg/mL. Infrared spectroscopy indicated that when UO_3_ is dissolved, UO_2_^2+^ is formed, while when UO_2_ is dissolved, there is no UO_2_^2+^ formed. The UO_2_ and UO_3_ were added to the DES, followed by ultrasonic treatment for approximately 3–4 h. For UO_2_, an additional heating step at 70 °C for 1 h was required to achieve complete dissolution after the ultrasonic treatment.

**Figure 1:**
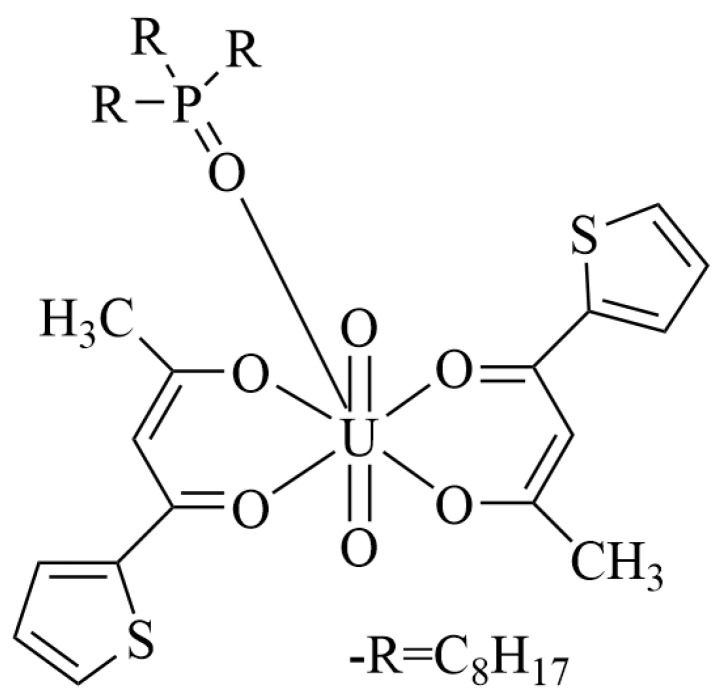
Possible coordination structure of uranyl ion in DES [32].

The HDESs provide an alternative dissolution pathway that is particularly useful for subsequent liquid–liquid extraction steps. UO_3_ can be completely dissolved at 80 °C within 1 h in the HDES (thenoyltrifluoroacetone (HTTA):trioctyl phosphine oxide (TOPO) = 2:1), and the solubility of UO_3_ is about 130 mg/mL (Figure 1) [32]. However, due to the lack of an oxidizing agent, U(IV) cannot be oxidized to UO_2_^2+^; thus, UO_2_ and U_3_O_8_ cannot be dissolved. This indicates that the effect of the DES varies with different compositions.

### 3.2. Dissolution of Plutonium Oxides

Building upon the success with uranium oxides, the application of DESs has been extended to the dissolution of other challenging actinide-bearing materials, most notably plutonium-containing matrices. The recovery of plutonium from refractory solids such as PuO_2_, mixed oxides (e.g., UO_2_ and PuO_2_), and contaminated waste forms is a critical yet difficult step in nuclear fuel cycle operations. Conventional methods necessitate aggressive treatments with hot concentrated nitric acid, often catalyzed by corrosive hydrofluoric acid, posing significant safety and environmental challenges.

The dissolution of PuO_2_ is widely recognized as a challenge. Interestingly, Kumar et al. [33] reported a groundbreaking acid-free strategy for plutonium recovery using a hydrophilic DES. A halide-free DES (choline citrate:urea = 1:2) was employed to leach plutonium from PuO_2_, UO_2_ and PuO_2_ mixed oxide, as well as simulated cellulosic waste loaded with PuO_2_ or Pu(NO_3_)_4_. Under controlled infrared heating at mild temperatures (~353 K), leaching efficiencies of ≥93% were achieved for all matrices, with the highest efficiency (97%) observed for nitrate-loaded waste. This process entirely avoids the use of mineral and hydrofluoric acids, significantly reducing corrosivity and secondary waste generation. The dissolved plutonium in the DES/propylene glycol system was subsequently subjected to selective solid-phase extraction (SPE) using extractant-encapsulated polymer beads, achieving a high distribution coefficient (K_d_ ≈ 950) and demonstrating notable selectivity for Pu(IV) over U(VI) and Am(III).

## 4. Extraction and Separation Processes

### 4.1. Extraction of Uranium

Extraction is a key process in industrial-scale actinide separation, exemplified by the PUREX (Plutonium-Uranium Reduction Extraction) process, which employs tributyl phosphate (TBP) in kerosene to extract U(VI) and Pu(IV) from nitric acid solutions. However, the use of volatile organic diluents and the generation of acidic waste streams are inherent drawbacks. DESs, particularly HDESs, have emerged as innovative, tunable media that can either directly replace organic phases or introduce new separation mechanisms.

**Figure 2:**
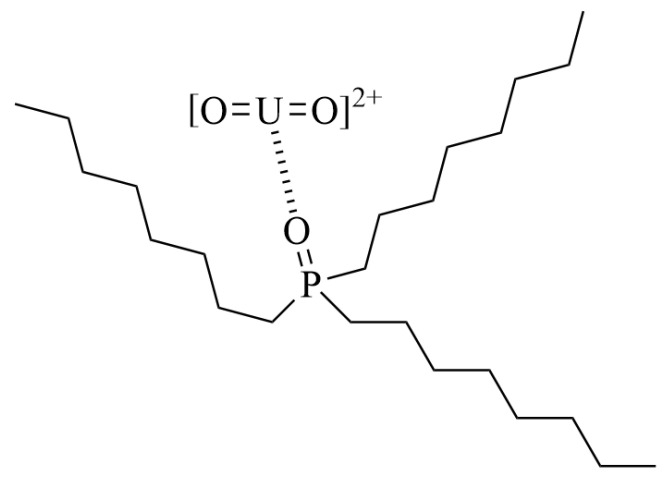
Combination of uranyl ions with TOPO in eutectic solvents [34].

TOPO is well-known as a neutral extractant for actinides, but its practical application is often limited by its low solubility in conventional hydrocarbon diluents and its narrow optimal acidity window. Thus, TOPO-based HDESs have been extensively investigated to overcome these limitations. A representative HDES (HTTA:TOPO) shows efficient extraction. At low acidity (0.5 M HNO_3_), U(VI), Pu(IV), Am(III), and Eu(III) can be extracted. At high acidity (>2 M HNO_3_), only U(VI) and Pu(IV) are effectively extracted, while the extraction efficiencies of Am(III) and Eu(III) decrease significantly. This enables selective separation at 5 M HNO_3_, achieving separation factors of ~30 for Pu(IV)/Am(III) and ~45 for U(VI)/Am(III). Loaded U and Pu can be efficiently stripped using 0.5 M ethylene diamine tetramethylene phosphonic acid (EDTMP), achieving recovery yields above 90% [32]. The HDESs (TOPO:menthol = 1:2–1:4) could be immobilized on diatomaceous silica-based solid support. The HDES (TOPO:menthol = 1:2) exhibited optimal performance, with a high loading (45%) and good thermal stability (up to 400 K). In 4 M HNO_3_ (25 °C, contact time of 60 min, liquid–solid ratio 50:1), this material showed a high uranium distribution coefficient (D = 1240) and an adsorption capacity of 19 mg/g. In particular, efficient and selective extraction of uranium (>98%) from industrial yellowcake was achieved, with effective suppression of competing metal ions including Fe(III), Mn(II), Ni(II), and Ce(III) (common impurities in uranium ore). The HDES and the diatomaceous silica maintained stable performance over five cycles and could be readily eluted with dilute nitric acid (Figure 2) [34]. TOPO was also combined with oxalic acid (OA) and MA to form two HDESs (DES-OA and DES-MA). Within 0.5–1.5 M HNO_3_ (25 °C, phase ratio (O/A) = 1:1, contact time of 30 min), both systems exhibited effective extraction of U(VI) and Am(III), with single-stage extraction efficiencies exceeding 90%. Spectroscopic analysis confirmed the formation of UO_2_(NO_3_)_2_(TOPO)_2_ complexes. Although their distribution coefficients are lower than those of conventional TOPO systems, these HDESs offer advantages in stripping efficiency [35]. To further broaden the applicable acidity range, the DES (TOPO:itaconic acid (IA)) was immobilized onto microporous polypropylene membranes. The resulting membrane exhibited stable uranium extraction efficiencies above 88% over a wide range of conditions (pH 1–9, 25 °C, contact time of 60 min or 1–7 M HNO_3_, 25 °C, contact time of 60 min). In 1–7 M HNO_3_ (25 °C, contact time of 60 min, liquid–solid ratio of 50:1), the extraction efficiencies reached ~95%, with distribution coefficients exceeding 2800 mL/g and a saturated adsorption capacity of 2500 μg/g [36]. While these TOPO-based HDES systems show promise as greener alternatives to TBP/kerosene, their relatively high viscosity and the need for further radiation stability data remain key challenges for large-scale deployment.

DESs also enable extraction strategies without inorganic acid, aligning with green chemistry principles. ChCl-organic acid DESs have been explored as simpler and metal-free alternatives for uranium extraction from solid matrices. A series of DESs synthesized from ChCl and lactic acid (LA) at different molar ratios were evaluated, and optimal extraction was achieved at a ChCl:LA:H_2_O ratio of 1:4:1. Under mild conditions (70 °C, 8 h, liquid–solid ratio of 10:1, atmospheric pressure), the uranium extraction efficiencies exceeded 95%. For minerals with known uranium contents (e.g., uranium-bearing ore samples with ~0.1% U content), recovery rates above 97% were obtained under the same conditions, with minimal co-extraction of competing ions such as Ca(II), Mg(II), Al(III), and Fe(III) (typical accompanying ions in geological uranium deposits). This indicates that this DES system has high efficiency and good selectivity for U(VI) relative to common earth-abundant metal ions [37].

To address the high viscosity of DESs and improve mass-transfer efficiency, a strategy to couple a DES with supercritical CO_2_ (SC CO_2_) was proposed. A halogen-free hydrophilic DES composed of choline dihydrogen citrate and urea (1:2) was developed, which is biodegradable, non-corrosive, and compatible with stainless steel SC CO_2_ equipment. Uranium compounds (UO_3_, UO_2_, magnesium diuranate and sodium diuranate) exhibited high solubilities in this DES (43–65 mg U/g DES) at 50 °C and atmospheric pressure. Subsequent extraction using SC CO_2_ (31 °C, 10 MPa, CO_2_ flow rate of 2 mL/min) with thenoyltrifluoro acetyl acetone (TTA) as a chelating agent achieved uranium recoveries of 45–65% [38].

### 4.2. Extraction of Plutonium

For plutonium, HDESs composed of quaternary ammonium/phosphonium salts and carboxylic acids (e.g., dodecyl triphenyl phosphonium bromide: decanoic acid) have shown remarkable selectivity for Pu(IV) in nitric acid. One such system achieved ~94% extraction of Pu(IV) in 5 M HNO_3_, while extraction of U(VI) and Am(III) was minimal [39]. This selectivity, coupled with good radiation stability (up to 600 kGy) and efficient stripping, highlights its potential for plutonium purification.

**Figure 3:**
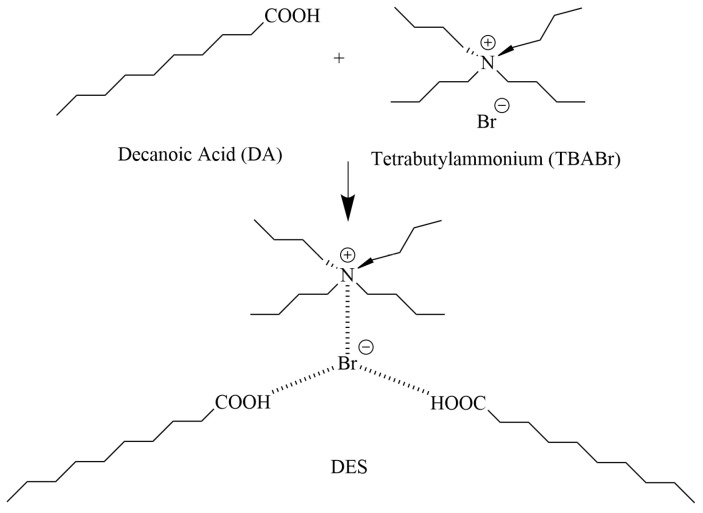
Structure of TBABR (upper left corner), DA (upper right corner) and DES [40].

Similar Pu-selective behavior was observed for other HDES systems. The HDES (tetrabutylammonium bromide:decanoic acid) enabled Pu(IV) extraction efficiencies of ~95% in 4 M HNO_3_ within short contact times (Figure 3). In addition, the HDES (undecanoic acid:tetraheptylammonium bromide) immobilized on a porous polypropylene membrane, exhibited strong and selective Pu(IV) adsorption (K_d_ ≈ 1000 mL/g) over a wide acidity range, with negligible uptake of U(VI) and Am(III) [40,41].

### 4.3. Extraction of Neptunium

**Figure 4:**
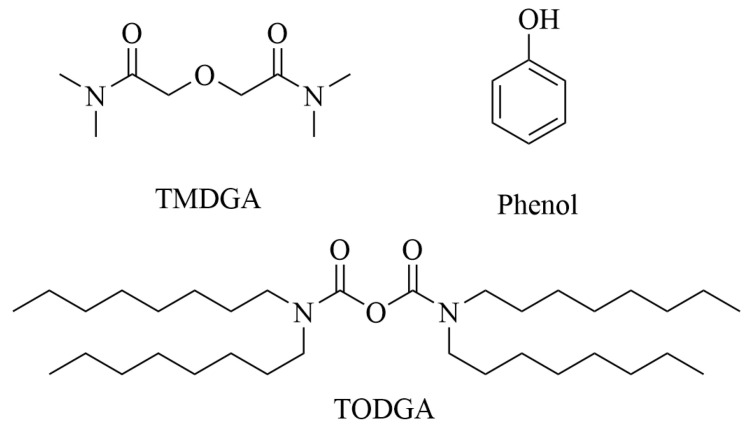
Chemical structures of TMDGA, phenol and TODGA [42].

**Figure 5:**
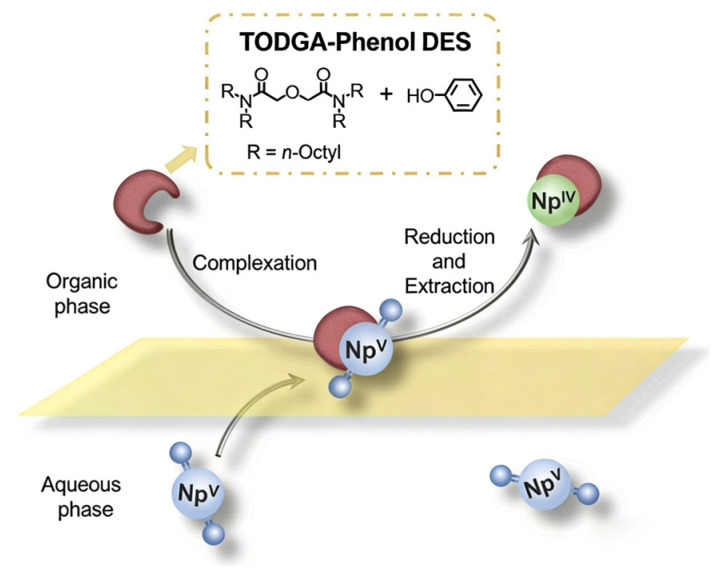
Efficient extraction of Np(IV) by TODGA-phenol DES [42].

For neptunium, whose pentavalent state (NpO_2_^+^) is notoriously difficult to extract in traditional systems, DESs enable a clever in situ redox-driven separation. An HDES composed of TODGA (N,N,N’,N’-tetraoctyl diglycolamide) and phenol was designed to extract Np(V) with >99% efficiency across a wide acidity range (0.001–2.0 M HNO_3_) (Figure 4) [42]. The mechanism involves a synergistic effect where phenol rapidly reduces Np(V) to Np(IV) within the aqueous phase, and the resulting Np(IV) is strongly complexed by TODGA and extracted. This process elegantly bypasses the need for external redox reagents and pre-adjustment steps required in aqueous-based flowsheets. These examples highlight the capability of DESs to control actinide oxidation states and leverage synergistic interactions to achieve superior selectivity (Figure 5).

In a distinct and highly efficient approach, the DES (TOPO:ortho-dihydroxybenzene) achieves a high distribution ratio (D_Np_ = 85) and an in situ reduction conversion rate of Np(V) to Np(IV) that exceeds 98% within 5 min over a broad nitric acid concentration range (10^−3^ to 2.0 M). Furthermore, the system exhibited robust stability under high salinity (up to 5 M NaNO_3_), a wide temperature range (293–313 K), and significant γ-irradiation doses (up to 60 kGy) [43].

### 4.4. Separation of Thorium

HDESs have also been explored for highly selective thorium separation and enrichment, motivated by the need to isolate Th from rare earth and alkaline earth elements. A series of HDESs were synthesized by combining 2-hexyldecanoic acid (HDA) as the HBD with various HBAs at appropriate molar ratios. These HDESs exhibited exceptional selectivity for Th(IV), with extraction efficiencies exceeding 98% (25 °C, 0.5 M HNO_3_, O/A = 1:1, contact time 30 min) and separation factors over 1000 relative to rare earth elements (La(III), Ce(III), Nd(III), Eu(III)) and alkaline earth ions (Ca(II), Sr(II)). The optimal thymol (TL):HDA (1:3) system showed a high thorium loading capacity (3.78 × 10^3^ mg/L) and allowed efficient stripping with dilute HCl, yielding a concentrated thorium solution with improved purity. The HDES maintained stable performance over multiple cycles, highlighting its potential for thorium recovery and resource utilization [44].

In the leaching solution of rare earth elements, efficient recycling of radioactive Th(IV) and U(VI) is equally important. An HDES (TOPO:N,N-dihexyl succinamic acid (DHSCA)) for the synergistic extraction of U(VI) and Th(IV) from sulfuric acid leachates of rare earth ores was examined. Within the pH range of 1.0–2.0, this HDES achieved removal efficiencies of >99% for both U(VI) and Th(IV) in a single-stage extraction while exhibiting excellent selectivity over Fe(III), Al(III), Mg(II), Ca(II), and various rare earth ions such as La(III) and Eu(III) (separation factors reaching over three orders of magnitude). Through a two-step stripping process (first with dilute sulfuric acid, then with ethylene diamine tetraacetic acid (EDTA) solution), effective separation and enrichment of U and Th were achieved. The HDES maintained stable extraction performance over seven consecutive cycles (Th(IV) > 98%, U(VI) > 97%). Slope analysis, ^31^P NMR, and FT-IR studies confirmed that hydrogen bonding between DHSCA and TOPO effectively shielded the P=O group of TOPO from coordinating with rare earth ions while enhancing the binding affinity of the system toward Th(IV) and U(IV), thereby enabling highly selective separation [45].

### 4.5. Separation of Actinides and Lanthanides

The separation of trivalent actinides (An(III)) from chemically similar trivalent lanthanides (Ln(III)) is one of the most challenging and critical steps in advanced spent nuclear fuel reprocessing, aimed at partitioning long-lived minor actinides for transmutation. Processes such as SANEX (Selective Actinide Extraction) rely on sophisticated soft-donor ligands, such as 6,6′-Bis(5,5,8,8-tetramethyl-5,6,7,8-tetrahydrobenzo-1,2,4-triazin-3-yl)-2,2′-bipyridine (CyMe_4_-BTBP), which exhibit higher affinity for An(III) over Ln(III). However, these systems often suffer from slow extraction kinetics and require optimization of the aqueous phase [46,47]. DESs offer novel avenues to enhance these separation processes, either as modifying co-solvents or as the primary extraction medium. Introducing DESs as co-solvents represents a promising strategy to overcome this limitation. Greta et al. [48] investigated four DESs based on choline acetate with different HBDs (urea, glycolic acid, diglycolic acid, imidazole) as co-solvents in the CyMe_4_-BTBP extraction system for Am(III), an actinide element that frequently coexists with lanthanides in nuclear waste streams and requires selective separation from lanthanides due to its radiotoxicity. Adding 5 vol% DES increased the Am distribution ratio (D_Am_) by ~four times compared to the control (D_Am_ = 2.88), with the DES (choline acetate:glycolic acid) showing the greatest enhancement (D_Am_ = 13.06). This DES also accelerated the extraction kinetics (30 min mixing time). The performance was stable at 5–20% DES content, but selectivity decreased above 35% due to enhanced Eu extraction. The DESs were structurally stable under irradiation. However, the study also noted that a high DES content (>35 vol%) could diminish the An(III)/Ln(III) selectivity by enhancing Eu(III) extraction. In this system, the extraction efficiency of simply physically mixing the HBA and HBD of the DES as the extractant is significantly lower than that when the formed DES is added to the system. This interesting observation may indicate that the dynamic stability of DESs as a whole is crucial for liquid extraction application, which needs further investigation.

### 4.6. Extraction of Fission Products

**Figure 6:**
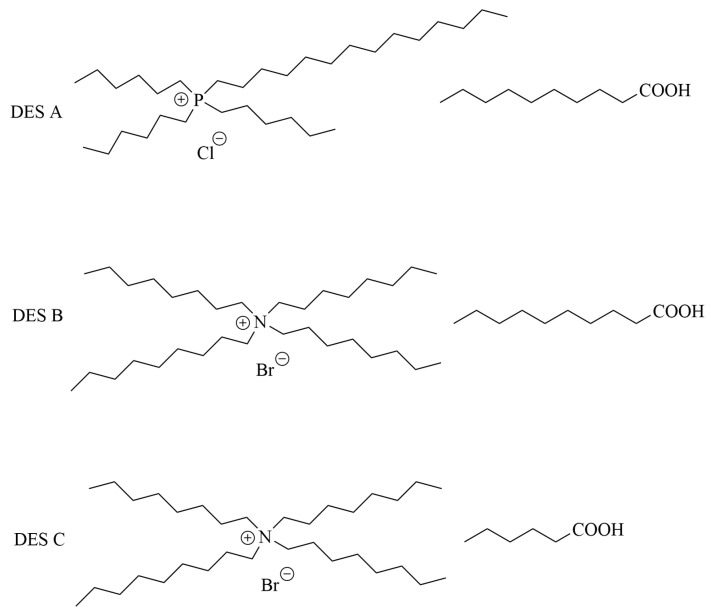
Three kinds of DESs for Tc extraction [49].

**Figure 7:**
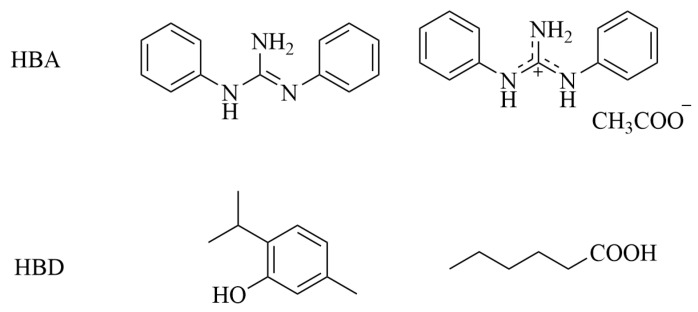
Components of DES for Tc extraction with radiation resistance [50].

Beyond the major actinides, the management of long-lived, mobile fission products is vital for safe waste disposal. ^99^Tc is a major long-lived fission product, and its anionic species TcO_4_^−^ exhibits high water solubility, chemical stability, and environmental mobility, posing a serious challenge for nuclear waste management. HDESs based on quaternary ammonium/phosphonium salts paired with fatty acids (e.g., trihexyltetradecylphosphonium chloride ([P_14,6,6,6_][Cl]) and decanoic acid) have demonstrated excellent affinity for TcO_4_^−^, achieving extraction efficiencies >99% and high distribution ratios (D_Tc_~10^3^–10^4^) from aqueous solutions (Figure 6) [49]. The extraction is believed to involve anion exchange or ion-pair formation. However, the selectivity can be compromised by competing anions such as ReO_4_^−^ and I^−^, especially under high dilution, indicating a potential limitation in complex waste streams. To improve robustness, radiation-resistant DES formulations have been explored. The DES (diphenylguanidine:hexanoic acid) maintained high Tc extraction efficiency (>98%) even after exposure to 250 kGy of γ-irradiation, highlighting its potential for direct application in radioactive environments (Figure 7) [50].

In addition, DESs (decanoic acid:diphosphonium salts with varied alkylene linkers) have been investigated for the extraction of TcO_4_^−^, U(VI), and Th (IV) from hydrochloric acid solutions. These DESs exhibited moderate extraction efficiency for U(VI) and Th(IV), with distribution ratios increasing with longer alkylene spacers in the diphosphonium cation. For TcO_4_^−^, the distribution ratios ranged from 4.2 to 20.3 depending on the linker length [51].

In the treatment of fission products, cesium-137 (^137^Cs) has attracted considerable attention due to its long half-life and environmental mobility. An example is the synthesis of a microporous silver selenidostannate in the DES (methylamine hydrochloride:N,N′-dimethylurea). This material exhibits exceptional selectivity for Cs^+^ over Sr^2+^, with distribution coefficients (K_d_) of 1.06 × 10^4^ mL/g and 87.7 mL/g, respectively, yielding a high separation factor (SF_Cs/Sr_) of ~121.4. In a continuous column experiment, AgSnSe^−1^ effectively processed 10,000 bed volumes of a mixed Cs^+^/Sr^2+^ solution (~6 ppm each), achieving removal rates of ~99.9% for Cs^+^ and nearly 0% for Sr^2+^. The exchanger also demonstrated excellent radiation resistance (up to 200 kGy β and 100 kGy γ) and stability across a broad pH range (1–12), highlighting the potential of DES-derived materials for efficient Cs^+^/Sr^2+^ separation in nuclear waste treatment [52].

A DES (lauric acid:thymol:camphor = 1:4:1) was prepared with addition of 10% bis(2-ethylhexyl) hydrogen phosphate (DEHPA) or 20% 18-crown-6 ether as an enhancer. The extraction efficiency of ^137^Cs was above 95% at pH 12 after only 1 min of vortexing (for DES with 10% DEHPA) or 8 min (for DES with 20% 18-crown-6 ether), achieving a 20-fold preconcentration and a detection limit of 0.40 Bq/mL. In particular, the method was successfully applied to the analysis of industrial wastewater, domestic sewage, and reservoir water samples, where the recovery efficiency of ^137^Cs was 95–98% with relative standard deviations below 6% [53].

A comparison across different systems suggests that ChCl-based DESs are most frequently employed (Table 2). These systems show promising performance for actinide dissolution and separation, as well as for the processing of selected fission products. However, the reported efficiencies are often achieved under laboratory-scale conditions. Despite these encouraging results, there are several common limitations. High viscosity remains a common challenge for many DES formulations, potentially restricting mass transfer and phase disengagement in practical processes. In addition, the long-term chemical and radiation stability of DESs under realistic nuclear fuel cycle conditions is still insufficiently explored. These observations indicate that the future development of DESs should focus not only on improving metal extraction or dissolution performance but also on addressing processing performance and stability issues to enable practical implementation. Additionally, the extraction efficiency and selectivity of DESs can vary significantly depending on their composition and the target metal ions. For instance, a comparative study by Shishov et al. demonstrated that for the extraction of rare earth elements from mining tailings, the efficiency of acidic DESs was lower than that of simple aqueous solutions of the corresponding acids, questioning the necessity of DESs in such specific contexts [54]. This suggests the need for continued optimization and a more in-depth mechanistic understanding.

## 5. Electrochemical Processing

Electrochemical processes are frequently involved in the nuclear fuel cycle, as they enable precise control over oxidation states and allow the direct deposition of metals or oxides. These processes are key steps in fuel fabrication, material recovery, and waste treatment. Conventional aqueous electrolytes are often unsuitable due to limited electrochemical stability, competing hydrogen evolution, and corrosion issues. DESs, with their wide electrochemical windows, good ionic conductivity, low volatility, and tunable coordination environment, have emerged as a promising new platform for electrochemical processing under mild, non-aqueous conditions.

### 5.1. Electrodeposition of Uranium

Conventional uranium electrodeposition methods suffer from intrinsic limitations, including low current efficiency, and significant secondary waste generation. Owing to their wide electrochemical window, good ionic conductivity, low volatility, and mild operating requirements, DESs have emerged as promising alternative media for uranium electrochemistry. Building on this advantage, successful uranium electrodeposition has been demonstrated in several DES systems. For example, a DES (ChCl:ethylene glycol = 1:2) that dissolved uranyl nitrate was used as an electrolyte to electrodeposit UO_2_ films on stainless steel, nickel, and copper substrates. Under optimized conditions (60 °C, 30 mA/cm^2^, 10 g/L UO_2_(NO_3_)_2_), the current efficiency was about 64.7%, significantly higher than that of aqueous systems, enabling low-temperature and non-aqueous uranium electrodeposition [55].

### 5.2. Electrochemical Redox Reaction

Beyond deposition performance, DESs also provide unique electrochemical behavior for uranium redox chemistry. In the DES (PTSA:ChCl = 1:2), U(VI) can be stably reduced to U(V) in a quasi-reversible single-electron process within a wide electrochemical window (~3.2 V), allowing potential-controlled uranium separation from coexisting metal ions. However, the high viscosity of this DES leads to slow uranium diffusion (1.87 × 10^−7^ cm^2^/s) and a low charge-transfer rate constant (3.76 × 10^−4^ cm/s), which may limit the deposition rates and require further optimization [29]. Similar kinetic features have been observed in other DES systems. In the DES (C_10_minBr:MA or C_10_minBr:diglycolic acid), the electrochemical windows exceed 2.6 V, but uranium reduction proceeds via an irreversible single-electron pathway with low diffusion coefficients (10^−8^–10^−10^ cm^2^/s), indicating mass-transfer constraints common to viscous DES electrolytes [31]. Understanding these structure–property relationships is crucial for designing DES electrolytes with tailored viscosity and conductivity for efficient electrodeposition.

### 5.3. Electrochemical Dissolution

Beyond deposition, DESs also facilitate controlled anodic dissolution and sensitive electrochemical detection of nuclear materials. In the DES (ChCl:ethylene glycol), metallic uranium undergoes anodic dissolution predominantly as U(IV), forming octahedrally coordinated complexes. Subsequent constant-potential electrolysis leads to partial oxidation to U(VI) and the formation of uranium-containing deposition films on the electrode surface, demonstrating the feasibility of integrated electrochemical processing in DES media [56]. In addition to electrodeposition, DES-based electrochemical systems have been applied to uranium analysis. After dissolving uranium matrices in the DES (ChCl:MA =1:1), differential pulse voltammetry enables direct quantification of uranium via its reduction current, with a detection limit as low as 0.079 × 10^−2^ mg/mL [30]. This method exemplifies how DESs can simplify sample preparation (by direct dissolution of solids) and enable rapid, on-site electrochemical analysis, offering an attractive alternative to complex instrumental techniques such as ICP-MS for certain monitoring applications.

## 6. Removal or Capture of Radioactive Nuclides

Beyond the core processes of dissolution and bulk separation, DESs have demonstrated significant utility in addressing critical challenges within the nuclear industry, including the remediation of contaminated surfaces. These applications leverage the green credentials, tunable chemistry, and multifunctionality of DESs to enhance operational safety and environmental protection.

The decontamination of radioactively contaminated surfaces (e.g., equipment, flooring, protective gear) is essential for personnel safety, facility maintenance, and waste minimization. A DES composed of heptyltriphenylphosphonium bromide and decanoic acid was incorporated into a poly (vinyl alcohol) matrix to form a peelable decontamination gel. At an optimal DES content of 20%, the gel achieved decontamination efficiencies of 99.5–99.9% for α- and γ-emitting nuclides (e.g., ^239^Pu and ^137^Cs) on various surfaces, significantly outperforming commercial detergent gels [57].

In another strategy, foam-assisted DES systems were developed to enable efficient decontamination across diverse materials and chemical environments. By combining different DES formulations with foam carriers containing surfactants, high decontamination efficiencies (>95%) were achieved for a wide range of radionuclides (U, Np, Pu, Th, Am, Cm, Ln, Sr, Cs, Tc) on various surfaces over a broad acidity range. In contrast, blank foam carriers exhibited much lower removal efficiencies, reflecting the dominant role of DES in radionuclide removal [18]. These DES-based systems provide efficient, broad-spectrum decontamination while reducing secondary liquid waste compared to traditional scrubbing methods.

To evaluate the potential of DESs for radioactive decontamination, using nonyl phenol polyoxyethylene ether (NP-10) as a surfactant, the DES (ChCl:PTSA = 1:2) was dispersed in SC CO_2_ to form a stable microemulsion system. Under optimized conditions (DES:NP-10 = 0.8:1, 333 K, 15 MPa, 75 min), the single-pass removal efficiency for UO_3_ contamination on stainless steel reached 89.0% and increased to 99.8% after a second treatment. This system showed broad applicability to different substrates (e.g., stainless steel, plastics, glass, ceramics) and radionuclides (U, Co, Cs, Eu, Sr, Th, Ce), with removal efficiencies generally exceeding 95%. For actual radioactive metal waste from the nuclear industry, the surface radioactivity was reduced to 0.09 Bq/cm^2^, well below the clearance limit (0.8 Bq/cm^2^) [17].

Further research addressing the performance limitations of this system proposed a novel strategy of adding hydrogen ion (H^+^) supplemental reagents to the microemulsions. Nitric acid was identified as an efficient and economical H^+^ supplement. Under optimized conditions (333 K, 15 MPa, 45 min), the DES in CO_2_ microemulsion system containing nitric acid achieved a decontamination efficiency of 99.0% for UO_3_ contamination on stainless steel surfaces, reducing the residual radioactivity below the exemption level. The technology demonstrated excellent versatility, with decontamination efficiencies exceeding 99.0% for uranium contamination on various substrates (stainless steel, plastic, glass, ceramic) and for multiple simulated radionuclides (U, Co, Mn, Cs, Eu, Sr, Th, Ce). More importantly, the system was successfully applied to real radioactive metal waste, achieving a decontamination efficiency of 99.0%, confirming its potential for industrial-scale application [58].

In the remediation of radioactively contaminated soil, DESs demonstrate significant potential as environmentally friendly solubilizing agents. Recently, Han et al. developed a composite leaching agent consisting of Na_2_CO_3_ (0.01 mol/L), NaHCO_3_ (0.025 mol/L), H_2_O_2_ (0.3 mol/L), and DES (ChCl:urea) (4 g/L) for the remediation of uranium-contaminated soil. Under the conditions of a solid–liquid ratio of 10:1 and a reaction time of 8 h, this system achieved a uranium removal rate of approximately 90% for actual contaminated soil, with pilot-scale tests reaching up to 92.6%. Mechanistic studies revealed that the Cl^−^ in the DES acts as a strong electron donor to coordinate with uranium, while urea disrupts U–O bonds, collectively promoting the dissolution of uranium from mineral lattices. Meanwhile, CO_3_^2−^ forms stable complexes with oxidized U(VI), facilitating its desorption from soil particle surfaces. This engineering application validates that the DES carbonate synergistic system enables efficient and mild remediation of uranium-contaminated soil [59].

Radioactive iodine (such as ^125^I, ^129^I and ^131^I) released from nuclear facilities is highly mobile and prone to biological accumulation, posing long-term environmental and health risks. Accordingly, recent studies have explored DES-based materials as green and efficient iodine-capturing media. One strategy focuses on the molecular-level design of DESs with strong iodine affinity. The DES (L-cysteine:LA = 1:8) achieved complete iodine removal within 7 h and exhibited a high capture capacity. Compared with previously reported iodine-absorbing DESs (e.g., iodinated choline-methylurea or polyethylene glycol -thiourea systems), this DES offers comparable efficiency while significantly reducing cost and improving biocompatibility and environmental friendliness. This work demonstrates that functional amino acids can endow DESs with strong iodine-binding capability while maintaining green chemistry advantages [60].

Beyond direct iodine capture, DESs have also been employed as pretreatment agents to construct multifunctional sorbents. A DES (urea:guanidine hydrochloride = 2:1) was used to pretreat silk fibers, producing a porous material with enhanced adsorption properties. After adsorbing organic dyes (especially methylene blue) from wastewater, the dye-loaded silk exhibited substantially improved iodine capture, reaching ~90% removal within 11 h—approximately twice that of untreated silk. In contrast, the DES-treated silk without dye loading showed limited iodine uptake [61]. The DES (choline iodide:N-methylurea) achieved nearly 100% I_2_ removal within 5 h, which was attributed to strong halogen bonding interactions. This system also exhibits superior iodine retention, with only 4.6% mass loss after 10 h under a nitrogen sweep, outperforming many conventional solvents and materials [62].

## 7. Detection

The rapid, sensitive, and selective detection of radionuclides in environmental and process streams is crucial for safety and safeguards. DESs contribute to this field by serving as advanced sensing media, modifying agents for nanomaterials, or matrices for sample pre-concentration.

Fluorescent probes based on DES-modified nanomaterials provide an attractive alternative. A ternary deep eutectic solvent (TDES) (ChCl:mercaptoacetic acid:thioacetate) was employed as a surface modifier for the one-step hydrothermal synthesis of CdSe quantum dots, avoiding inert atmospheres and toxic reagents. The resulting TDES-modified CdSe quantum dots (QDs), semiconductor nanocrystals with size-dependent optical and electronic properties arising from quantum confinement effects, exhibited high selectivity and sensitivity toward UO_2_^2+^, with a linear detection range of 10–500 nM, a detection limit of 5.7 nM, and a response time below 5 min. Interference from Cu^2+^ could be effectively suppressed by thiourea addition, while common cations and anions showed negligible effects. Langmuir fitting indicated monodentate binding of UO_2_^2+^ on the QD surface at low concentrations [63]. Conversely, the DESs themselves can be engineered as superior luminescent media. In addition, highly selective HDES-based photosensitive sensing membranes have been developed. The HDES (TOPO:decanoic acid = 1:1) was incorporated into a cellulose triacetate film containing a colorimetric indicator. The membrane exhibited a visible color change upon uranium binding and allowed quantitative detection by colorimetry (the detection limit was 0.084 × 10^−6^ M; the linear range was 0.84–8.4 × 10^−5^ M) or X-ray fluorescence (the linear detection range of uranium ions was 0.021–2.1 × 10^−3^ M) without matrix separation. Uranium in real seawater samples was directly determined at ~10^−8^ M, and the membrane could be regenerated and reused multiple times with stable performance [64]. For high-precision isotopic analysis, DESs have been coupled with thermal ionization mass spectrometry (TIMS). A DES (TOPO:IA = 0.6:0.4) immobilized on a polypropylene membrane enables efficient uranium extraction, and the membrane decomposes completely upon heating on the TIMS filament without residue. This approach simplifies sample preparation and minimizes uranium loss and contamination. At the ppb level, isotope ratio measurements in synthetic groundwater, urine, seawater, and groundwater matrices showed a reproducibility of ~10% relative standard deviation, demonstrating suitability for trace and ultra-trace uranium analysis [36]. The morphology of Pd nanoparticles has an impact on the detection effect. By adjusting the moisture content and temperature of the DES, the morphology of the Pd nanoparticles could be effectively controlled, reducing particle aggregation and forming a high surface area electrode interface. Pd nanoparticles were electrodeposited in the DES (ChCl:urea = 1:2). This interface enabled U(VI) detection by differential pulse voltammetry with a detection limit of 3.04 nM and excellent selectivity for UO_2_^2+^ [65].

To develop a rapid, sensitive, and cost-effective approach for thorium detection in complex environmental matrices, an HDES composed of 1-hexyl-3-methylimidazolium bromide ([HMIM]Br) and salicylic acid (SA) was designed as an extractant. By introducing thorin and cetyltrimethylammonium bromide (CTAB) to form a Th(IV)-thorin-CTAB ternary ion-pair complex, efficient enrichment of Th(IV) into the HDES phase was achieved. Under optimized conditions, the method exhibited a linear detection range of 10–600 ng/mL, a low detection limit of 2.1 ng/mL, and good precision (relative standard deviation, 1.7%). Common interfering ions could be effectively masked, and the method was successfully applied to river water, seawater, and rock samples, demonstrating its practical applicability for trace Th(IV) analysis [66].

## 8. Adsorption

The development of nuclear energy and uranium-related industries involves the discharge of large amounts of uranium-containing wastewater, which is mainly derived from uranium ore mining and refining, nuclear fuel processing (e.g., uranium conversion and enrichment), and radioactive waste treatment processes [1,67]. Uranium-containing wastewater poses potential risks to ecological environments and human health due to its radiotoxicity and bioaccumulation. Therefore, there is an urgent need to develop efficient, low-cost and environmentally friendly uranium adsorption materials.

The traditional hydrothermal carbonization (HTC) surface has poor functional groups and limited adsorption capacity. It was demonstrated that DESs can be used for adsorption material preparation. Cotton straw was mixed with a DES (ChCl:citric acid (CA) = 1:1) at 180 °C to obtain solvothermal carbon 180 (STC180), which exhibited the highest adsorption capacity of 353 mg/g for U(VI) and reached adsorption equilibrium within 2 h. Its adsorption performance was stable within the pH range of 4.0–8.0. It exhibits certain competitive adsorption toward Ca^2+^, meaning Ca^2+^ can occupy a portion of the active adsorption sites on STC180 that would otherwise bind to U(VI). However, this competitive effect is mild. Even in aqueous solutions containing 50 mg/L Ca^2+^ (a concentration comparable to that in natural groundwater or industrial wastewater), the U(VI) adsorption capacity of STC180 only decreases by ~8–10%, remaining above 318 mg/g. This indicates that the material maintains high U(VI) adsorption efficiency despite the presence of Ca^2+^. After five adsorption–desorption cycles, the adsorption performance remained stable [67].

Adsorbent materials can be prepared by biomass (such as cellulose, bamboo powder, straw, etc.) in DES (allyl trimethyl ammonium chloride:OA = 1:1) and then copolymerized with vinylphosphonic acid to introduce phosphate groups. It was found that the prepared phosphated biomass adsorbent had a maximum adsorption capacity of about 886 mg/g at pH = 3.0. Even under strong acid conditions of 1.0 M HNO_3_, it maintained an adsorption capacity of about 186 mg/g. Moreover, it reached the adsorption equilibrium within 30 s. For the real uranium tailings wastewater (pH = 3.23, initial uranium concentration 0.878 mg/L), the uranium concentration was reduced to 18 μg/L after being treated with adsorption, which was much lower than the national emission standard (50 μg/L) and could be directly discharged [68].

## 9. Nuclear Fuel Preparation

The traditional preparation of UO_2_ fuel microspheres mainly relies on the sol-gel method, which is a cumbersome process and suffers from limitations such as uneven particle size control and poor product uniformity. The use of DESs can simplify the process and improve the material uniformity.

More recently, “actinide-type” DESs have been developed by directly combining uranyl nitrate hexahydrate (UNH) and urea. At an optimal molar ratio of 0.2:0.8, a stable eutectic liquid is formed at room temperature with a melting point of −5.2 °C. Uranium exists as UO_2_^2+^ in the DES, and controlled electrodeposition yields aggregated UO_2_ nanoparticles with sizes of 30–40 nm, highlighting the potential of actinide-based DESs for uranium electro-synthesis [69,70]. Our group has proposed a method for nuclear fuel fabrication. The DES (UNH:urea = 1:4 or 1:9) forms droplets by vibrating nozzles or microfluidic technology. After solidification with aqueous ammonia, the process involves preheating, heating reduction (at 680–900 °C) and high-temperature sintering (at 1500 °C), resulting in uniformly sized (adjustable from 10 to 700 μm) UO_2_ microspheres. This method requires fewer reagents and has a high uranium content, making it more suitable for large-scale production [71]. In the preparation of mixed oxide (MOX) fuel, the traditional wet process is lengthy and generates a large amount of waste liquid, while the dry process is complex in operation. It was found that nitrate or oxalate salts could form DESs with urea; thus, an even mixture at the molecule level is possible. By forming DESs through the reaction of nitrate or oxalate salts of uranium, thorium, and plutonium with urea, after removing urea through heating, and then decomposing the nitrate or oxalate salts at a higher temperature, uniform and controllable-sized AnO_2_-type MOX powder can be directly obtained. There are no dissolution or precipitation steps required, and very little waste liquid is produced [72].

## 10. Green Metrics and Technoeconomic Considerations

The features of DESs can reduce solvent loss, energy consumption, and secondary waste generation, which is particularly important in radiochemical processes requiring strict containment and safety control. However, the environmental effects of DESs depend on their composition and application [73]. Certain HDESs based on long-chain organic components may exhibit limited biodegradability or high toxicity. From a technoeconomic standpoint, DESs are attractive due to their low raw material cost and their ability to integrate dissolution, redox control, extraction, or electrochemical processing within a single medium, potentially reducing process complexity. At present, green chemistry metrics and life cycle assessment approaches have been increasingly applied to quantitatively evaluate the environmental impact of DESs. For example, Pishro et al. reported that the analytical greenness metric score for caffeine extraction using an HDES reached 0.83, which was significantly higher than the values obtained with conventional organic solvents (0.15–0.25) [74]. Nevertheless, in specific systems, different conclusions may be obtained when considering the entire life cycle. For instance, Bouhzam et al. investigated the extraction of polyphenols from spent coffee grounds and demonstrated through life cycle assessment that, even after process optimization and partial solvent recovery, certain DES systems may still exhibit higher environmental burdens due to factors such as the production of their constituent components and energy consumption during operation [75]. These findings indicate that the environmental impact of DESs is strongly dependent on their specific compositions and should be evaluated through a comprehensive assessment combining green analytical metrics with life cycle analysis. From a practical perspective, high viscosity, mass-transfer limitations, and uncertainties regarding long-term radiolytic stability remain key challenges that may increase operational costs at larger scales [76]. Overall, while DESs show clear potential as greener and economically viable alternatives, their practical implementation in the nuclear fuel cycle requires case-by-case assessment and further optimization [77].

## 11. Conclusions and Perspectives

Despite the promising results demonstrated by DESs in various aspects of the nuclear fuel cycle, including dissolution, extraction and redox reactions etc., their research and application in this field are still at an early and rapidly developing stage. Current studies are concentrated in only a few countries, and a more extensive and systematic international research network is highly desirable. Additionally, there are key challenges remaining to be addressed. The high viscosity of DESs significantly impacts mass transfer and process efficiency. Although it is known that viscosity can be adjusted by modifying components or adding water, there is currently a lack of systematic theoretical models to guide theoretical design. Mechanisms of the chemical processes in DESs remain unclear, which constrains their theoretical design and optimization. In addition, research on the behavior of DESs under strong radiation fields remains relatively limited. The industrial application of DESs is extremely difficult at present for the back end, for example, the PUREX process and the treatment of high-level liquid waste (HLLW), due to the high radioactivity and complex composition involved. For the front end of the nuclear fuel cycle, for example, mining and fuel preparation, DESs show great promise for being applied industrially due to the relatively low radioactivity levels.

## Figures and Tables

**Table 1:** DES classification table [8,11,28].

Classification Type	Examples of Hydrogen Bond Acceptors (HBAs)	Examples of Hydrogen Bond Donors (HBDs)	Main Characteristics
Type I	Quaternary ammonium salts (e.g., ChCl)	Metal chloride (e.g., ZnCl_2_)	Early-developed, hygroscopic, moisture-sensitive.
Type II	Quaternary ammonium salts	Hydrated metal salts (e.g., CrCl_3_·6H_2_O)	Contain crystal water, hydrogen-bond network influenced by hydration.
Type III	Quaternary ammonium salts	Organic compounds (e.g., urea, glycerol, sugar)	Most common, structurally diverse, widely used in separation and extraction.
Type IV	Metal salts (e.g., MgCl_2_·6H_2_O)	Organic compounds (e.g., glycerol and acetamide)	Metal ions participate in coordination, suitable for electrochemical and radiochemical applications.
Type V	Nonionic compounds (e.g., menthol and thymol)	Nonionic compounds (e.g., carboxylic acids)	Hydrophobic system for non-polar extraction and biphasic separation.

**Table 2:** Extraction efficiency of actinides.

Composition of DES	Extracted Substances	Extraction Efficiency	Interfering Ions	Extraction Conditions	References
HTTA:TOPO (2:1)	U(VI), Pu(IV), Am(III), Eu(III)	separation factor of ~30 for Pu(IV)/Am(III) and ~45 for U(VI)/Am(III)	-	5 M HNO_3_	[32]
TOPO:menthol (1:2) (immobilized on diatomaceous silica)	U(VI)	>98%	Fe(III), Mn(II), Ni(II), Ce(III)	4 M HNO_3_, 25 °C, contact time 60 min, liquid–solid ratio 50:1	[34]
TOPO:OA/TOPO:MA	U(VI), Am(III)	>90%	-	0.5–1.5 M HNO_3_, 25 °C, phase ratio (O/A) = 1:1, contact time 30 min	[35]
TOPO:IA (immobilized on polypropylene membrane)	U(VI)	Extraction efficiency >88% (pH 1–9); ~95% extraction efficiency in 1–7 M HNO_3_	-	25 °C, contact time 60 min, liquid–solid ratio 50:1	[36]
ChCl:LA:H_2_O (1:4:1)	U(VI)	Extraction efficiency >95% for U(VI) from uranium ore; recovery rate >97% for samples with known uranium content	Ca(II), Mg(II), Al(III), Fe(III)	70 °C, 8 h, liquid–solid ratio 10:1, atmospheric pressure	[37]
Dodecyl triphenyl phosphonium bromide:decanoic acid	Pu(IV)	~94%	U(VI), Am(III)	-	[39]
Tetrabutylammonium bromide:decanoic acid	Pu(IV)	~95%	U(VI), Am(III)	Short contact time	[40]
Undecanoic acid:tetraheptylammonium bromide (immobilized on polypropylene membrane)	Pu(IV)	>99%	U(VI), Am(III)	Wide acidity range	[41]
TODGA:phenol	Np(V)	>99%	-	0.001–2.0 M HNO_3_	[42]
TOPO:ortho-dihydroxybenzene	Np(V)	>98%	-		[43]
HDA with various HBA (e.g., TL) (1:3)	Th(IV)	>98%	La(III), Ce(III), Nd(III), Eu(III), Ca(II), Sr(II)	10^−3^–2.0 M HNO_3_, 293–313 K, contact time 5 min	[44]
TOPO:DHSCA	U(VI), Th(IV)	>99%	Fe(III), Al(III), Mg(II), Ca(II), La(III), Eu(III)	25 °C, 0.5 M HNO_3_, phase ratio (O/A) = 1:1, contact time 30 min	[45]

## Data Availability

No new data were created or analyzed in this study. Data sharing is not applicable to this article.
